# Efficacy and safety of carbon‐ion radiotherapy for the malignant melanoma: A systematic review

**DOI:** 10.1002/cam4.3134

**Published:** 2020-06-10

**Authors:** Chengcheng Li, Qiuning Zhang, Zheng Li, Shuangwu Feng, Hongtao Luo, Ruifeng Liu, Lina Wang, Yichao Geng, Xueshan Zhao, Zhen Yang, Qiang Li, Kehu Yang, Xiaohu Wang

**Affiliations:** ^1^ The First School of Clinical Medicine Lanzhou University Lanzhou China; ^2^ Institute of Modern Physics Chinese Academy of Sciences Lanzhou China; ^3^ Lanzhou Heavy Ions Hospital Lanzhou China; ^4^ Basic Medical College Lanzhou University Lanzhou China; ^5^ Evidence‐Based Medicine Center School of Basic Medical Sciences Lanzhou University Lanzhou China

**Keywords:** carbon‐ion radiotherapy, malignant melanoma, systematic review

## Abstract

Malignant melanomas (MMs) were the fifth most common cancer in men and the sixth most common cancer in women in 2018, respectively. These are characterized by high metastatic rates and poor prognoses. We systematically reviewed safety and efficacy of carbon‐ion radiotherapy (CIRT) for treating MMs. Eleven studies were eligible for review, and the data showed that MM patients showed better local control with low recurrence and mild toxicities after CIRT. Survival rates were slightly higher in patients with cutaneous or uveal MMs than in those with mucosal MMs. CIRT in combination with chemotherapy produced higher progression‐free survival rates than CIRT only. In younger patients, higher rates of distant metastases of gynecological MMs were observed. The data indicated that CIRT is effective and safe for treating MMs; however, a combination with systemic therapy is recommended to ensure the best possible prognosis for MMs.

## Graphical abstract

The purpose of this study is to systematically review the safety and efficacy of carbon‐ion radiotherapy (CIRT) for malignant melanoma. Eleven studies were eligible. For overall survival, cutaneous melanoma, and uveal melanoma had a slight advantage than mucosal melanoma. Concurrent CIRT with chemotherapy exhibited superior PFS than CIRT alone. Younger patients presented a higher distant metastasis for gynecological melanoma. Although available data demonstrated that malignant melanoma is effectively and safely treated with CIRT, it is suitable to organically combine systemic therapy to obtain the best prognosis for melanoma patients.

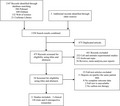

## 1 INTRODUCTION

Malignant melanomas (MMs) originate from melanocytes in the basal layer of the epidermis, and they are characterized by high malignancy, early metastasis, poor prognosis, and high mortality. Depending on their location, MMs can be classified as cutaneous, uveal or mucosal. Previous studies suggest that average morbidity rates are 16.2 per 100 000 in the United States and 0.383 per 100 000 in Japan.^1^ However, MMs incidence increased continuously worldwide.^2^, ^3^ MMs were the fifth most common cancer in men and the sixth most common cancer in women in 2018. According to the American Association of Cancer (AJCC) TNM staging system, the 5‐year overall survival (5‐y OS) rates are approximately 98.2% in patients at stages I/II, 61.7% at stage III, and 15.2% at stage IV.^4^ Although stage I MMs are typically associated with good prognoses after surgical treatment only, achieving negative margins in all patients was difficult. Currently available treatment options for patients with MM include radiotherapy (RT), chemotherapy, targeted therapy, and immunotherapy. Adjuvant therapies were recommended as an option for patients who were diagnosed with unresectable or advanced forms of MM. However, conventional chemotherapy regimens showed low response rates and produced considerable adverse effects.^5^ Immunotherapy‐related studies require following up over long periods of time.^6^, ^7^ The European consensus‐based interdisciplinary guidelines suggest RT for cases in which surgery would lead to severe disfigurement or if tumors or metastases are inoperable, particularly regarding patients with tumors at stages III or IV.^8^ Melanoma cells are typically resistant to RT owing to their high capacity of sublethal damage repair; therefore, applicability of this form of treatment is largely limited to adjuvant therapy after surgical resection or to patients refusing surgery. With the development of imaging and radiation techniques, RT has become an important adjuvant therapy for advanced or metastatic MMs.

Carbon‐ion beams produce increased energy deposition at the end of their range to form the Bragg peak while minimizing irradiation damage to the surrounding tissues, which facilitates precise dosage and localization, compared with photon beams. Furthermore, carbon‐ion beams are cell cycle independent with a high relative biologic effectiveness (RBE) and low oxygen enhancement ratios.^9^ This kind of radiation also leads to double‐strand breaks in DNA molecules, resulting in lethal damage to tumor cells. These properties are thus advantageous for treating radiation‐insensitive tumors. RT using carbon‐ion beams could be a valuable technology for treating MMs to decrease treatment‐related toxicity and to improve therapeutic effects. In 1984, the first heavy‐ion medical accelerator for cancer therapy was constructed in Japan. Clinical trials of carbon‐ion therapy commenced at the Japanese National Institute of Radiological Sciences (NIRS) in 1994.^10^ Since then, carbon‐ion RT (CIRT) has been used for treatments of various kinds of tumors, especially for radio‐resistant tumors and tumors situated near important organs. Currently, the use of CIRT for treating MMs has been reported in Japan, Italy, Germany, and China at seven institutions: NIRS, Hyogo Ion Beam Medical Center (HIBMC), Gunma University Heavy Ion Medical Center, SAGA‐HIMAT Foundation, National Center of Oncological Hadrontherapy, Heidelberg Ion Beam Therapy Center and Heavy Ion Research Facility, Lanzhou, Institute of Modern Physics. It is important to closely assess whether CIRT is superior over other therapeutic options for treating MMs. We therefore systematically reviewed the currently available data to comprehensively examine clinical efficacy and safety of CIRT for treating MMs.

## 2 MATERIALS AND METHODS

### 2.1 Search strategy

This systematic review was conducted according to the Preferred Reporting Items for Systematic Reviews and Meta‐Analyses (PRISMA) statement, and the review protocol is available in PROSPERO (CRD42019141495). All literature searches were conducted until October 15, 2019, using the search tools of Embase, Cochrane Library, Web of Science, and PubMed with the search terms “hadron”, “particle”, “charged particle”, “heavy ion*”, OR “carbon ion”, AND “melanoma”. In order to include as many eligible studies as possible, one last duplicate search was performed on March 5, 2020. Only publications written in English were considered. Additional studies were identified from citations in conference abstracts, review articles, and reference lists. All references were screened to ensure that relevant studies were included.

### 2.2 Inclusion and exclusion criteria

Studies were included if they matched the following criteria: (a) clinical or retrospective studies reporting effectiveness and/or adverse effects on patients with MMs who were treated with carbon‐ion beams; (b) Trials enrolling adults; and (c) tumors had been diagnosed by histopathology. Publications were excluded if they were (a) studies only reporting photons, protons or other heavy particles; (b) case reports on one or two patients; (c) letters, editorials, protocols, reviews, or meta‐analyses; (d) duplicate publications; (e) cell and animal experimental studies; (f) lacking detailed data.

### 2.3 Data extraction

The necessary data was extracted by two investigators independently, and results were discussed with senior investigators until a consensus was reached. The primary outcome was OS, and secondary outcomes were local control (LC), progression‐free survival (PFS), treatment‐related toxicity, local recurrence, and distant metastases (DM). The following data of each article were processed: first author, publication year, study design, number of patients, institution, study period, tumor site, tumor status, total treatment dose, and median follow‐up time.

### 2.4 Quality assessment

Case series reports were evaluated using the case series report bias evaluation tool in Table 1.^11^ With yes, no, and unclear. Given that the evaluation tool with 23 items is too cumbersome, this paper is streamlined to eight. The evaluation indicators follow: (a) Inclusion criteria and exclusion criteria; (b) Clinical heterogeneity of patients, including the severity, classification, duration, and onset time of the disease; (c) Whether the main intervention measures are clearly described (dose, administration, and course of treatment, etc); (d) Whether the measurement method of relevant outcome measures is reasonable; (e) Whether the outcome measures are measured before and after the intervention; (f) Whether the loss to follow‐up and follow‐up time are reported; (g) Whether the occurrence of adverse events related to clinical treatment is reported; (h) Whether the outcome measurer is blinded. Literature quality evaluation was independently completed by two members, respectively.

**TABLE 1:** Case series report quality evaluation form

Studies	1	2	3	4	5	6	7	8
Murata et al	No	Yes	Yes	Yes	Unapplicable	Yes	Yes	Unapplicable
Barcellini et al	No	Yes	Yes	Yes	Unapplicable	Yes	Yes	Unapplicable
Mohr et al	No	Yes	Yes	Yes	Unapplicable	Yes	Yes	Unapplicable
Demizu et al	Yes	Yes	Yes	Yes	Unapplicable	Yes	Yes	Unapplicable
Kagawa et al	No clear	Yes	Yes	Yes	Unapplicable	Yes	Yes	Unapplicable
Zhang et al	Yes	No	Yes	Yes	Unapplicable	Yes	No clear	Unapplicable
Mizoe et al^12^	No	No	Yes	Yes	Unapplicable	Yes	No clear	Unapplicable
Mizoe et al^13^	Yes	No clear	Yes	Yes	Unapplicable	Yes	No clear	Unapplicable
Toyama et al	No	Yes	Yes	Yes	Unapplicable	Yes	Yes	Unapplicable
Shirai et al	No	Yes	Yes	Yes	Unapplicable	Yes	No	Unapplicable
Vitolo et al	No clear	No clear	Yes	Yes	Unapplicable	Yes	Yes	Unapplicable

## 3 RESULTS

### 3.1 Study characteristics

**FIGURE 1:**
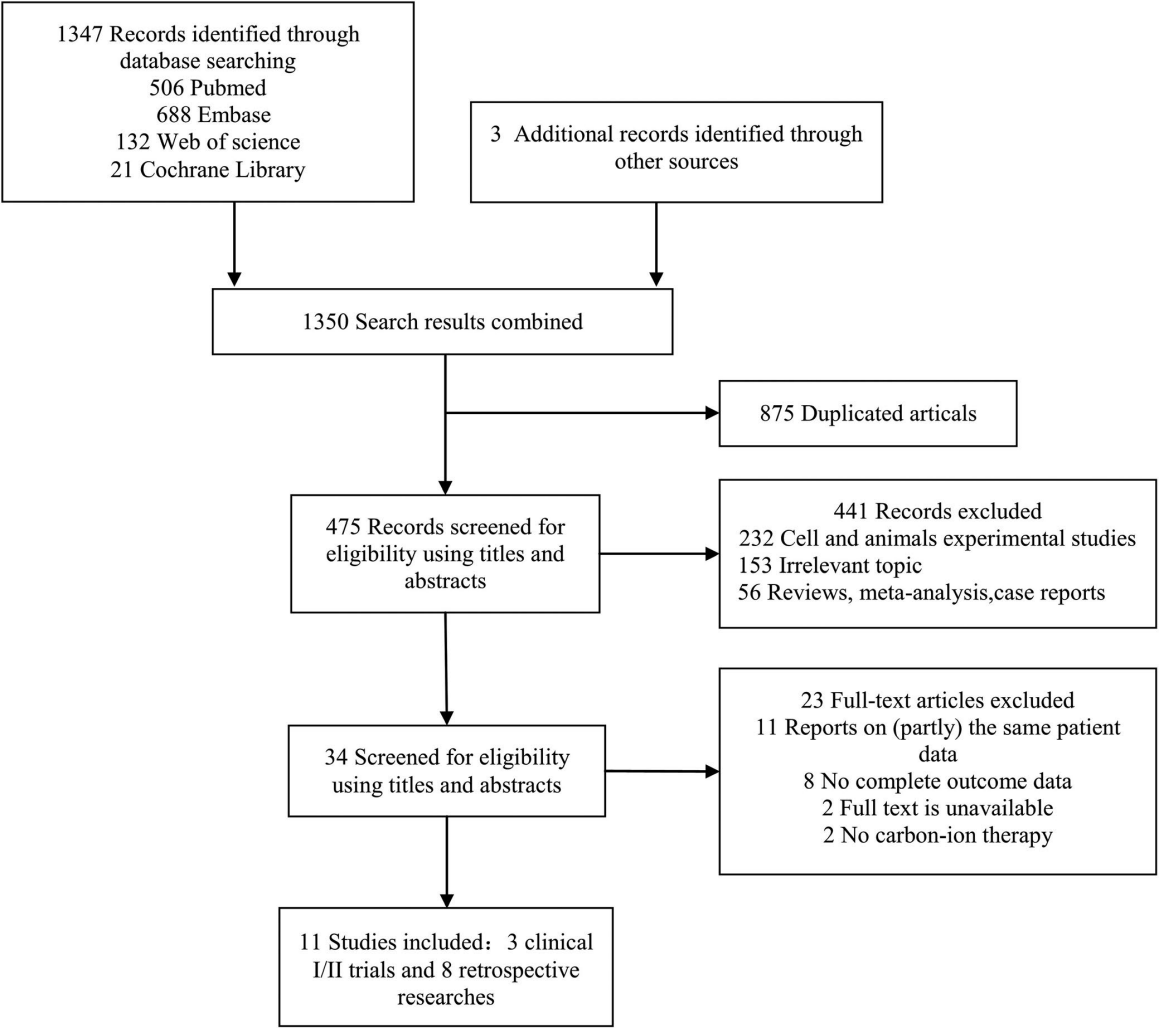
Flow chart depicting literature search

As shown in Figure 1, the searches produced a total of 1350 publications, of which 1316 reports were removed as based on skimming titles and abstracts. After full‐text review, we removed another 23 items, including 11 reports on (partly) the same patients' data, 8 publications with incomplete outcome data, 2 studies in which no full text was available, and 2 studies that did not include CIRT. Eleven studies (three clinical stage‐I/II trials and eight retrospective studies) with data of 360 patients were included and the relevant data were extracted.^12^, ^13^, ^14^, ^15^, ^16^, ^17^, ^18^, ^19^, ^20^, ^21^, ^22^ The main characteristics are shown in Table 2. Sample size ranged from 4 to 116 patients, and median follow‐up time ranged from 8 to 90 months. The studies included cutaneous, uveal, and mucosal MMs. Two clinical and two retrospective studies had been conducted at the NIRS,^12^, ^13^, ^15^, ^22^ two retrospective studies at the HIBMC,^14^, ^16^ one retrospective study at the GHMC in Japan,^18^ and two retrospective studies originated from Italy.^17^, ^21^ The only study on cutaneous MMs originated from China,^20^ and one clinical trial from Germany reported paranasal sinus MMs.^19^ The median age of patients ranged from 56 to 72 years. At least five studies included recurrent cases, and at least one study dealt with patients with DM.

**TABLE 2:** Baseline characteristics of included studies

Study	Country	Study type	Time range	No. of patients	Tumor site	Follow‐up (month)
Mizoe et al^12^	Japan (NIRS)	Prospective	1994.06‐1997.01	5	H&N (mucosa)	90 m (77‐108)
Kagawa et al^14^	Japan (HIBMC)	Retrospective	2002.02‐2002.07	8	H&N (mucosa)	24 m (5‐31)
Mizoe et al^13^	Japan (NIRS)	Prospective	1997.04‐2006.02	85	H&N (mucosa)	54 m (3‐162)
Toyama et al^15^	Japan (NIRS)	Retrospective	2001.01‐2012.02	116	Choroid	55.2 m (6‐127)
Demizu et al^16^	Japan (HIBMC)	Retrospective	2003.10‐2011.04	CIRT: 29 Proton RT: 33	H&N (mucosa)	25.9 m (5.2‐82.7)
Vitolo et al^17^	Italy (CNAO)	Retrospective	2013.5‐2014‐10	8	H&N (mucosa)	8 m (3‐12)
Shirai et al^18^	Japan (GHMC)	Retrospective	2011.06‐2016.12	43	H&N (mucosa)	26 m
Mohr et al^19^	Germany	Retrospective	2009.12‐2013.08	18	Paranasal sinus (mucosa)	18 m (5‐48)
Zhang et al^20^	China	Prospective	2006.11‐2009.03	7	Skin	24 m (14‐36)
Barcellini et al^21^	Italy	Retrospective	2016.01‐2017.02	4	Female genital organs (mucosa)	NR
Murata et al^22^	Japan (NIRS)	Retrospective	2004.01‐2017‐12	37	Female genital organs (mucosa)	23 m (5‐103)

Abbreviations: CNAO, National Center of Oncological Hadrontherapy; GHMC, Gunma University Heavy Ion Medical Center; HIBMC, Hyogo Ion Beam Medical Center; NIRS, National Institute of Radiological Sciences; NR, no reported.

### 3.2 Comparing treatment efficacy of CIRT and MMs

Data regarding tumor status, treatment planning, OS, LC, other therapies, local recurrence, and DM are shown in Table 3. Only one clinical trial conducted in China assessed associations between CIRT only and cutaneous MMs. The 1‐y OS and LC rates were 71.4% and 71.4%, respectively, and 3‐y OS and LC rates were 71.4% and 42.9%, respectively. Two patients (28.6%) showed local recurrences, and one of them developed DM.^20^ A different study examined CIRT for treating choroidal MMs, with 5‐y OS and, 5‐y LC rates of 80.4% and 92.8%, respectively, and local recurrence and DM rates of 5.3% and 25.1%, respectively.^15^ One study produced in Italy and five studies conducted in Japan reported associations between CIRT and head and neck (H&N) MMs. A dose escalation study conducted at NIRS for the first time reported 5‐y LC rates of five patients in 2004; however, the number of cases may have been too low, resulting in a 5‐y LC rate of 100%. The authors concluded that dose fractionation of 70.2 Gy (RBE)/18 fractions/6 weeks and 64.0 Gy (RBE)/16 fractions/4 weeks produced equal morbidity and local control.^12^ Until 2012, 85 participants were subjected to the same treatment plan in a second clinical trial. Considering that extensive skin areas were targeted, the effective total dose was reduced to 57.6 Gy (RBE). Both publications did not elaborate on tumor stage or size. Five‐year OS and LC were 35% and 75%, respectively. Recurrence was observed in 2.4% of the patients, and 41% showed DM.^13^ At a different Japanese institution, eight patients diagnosed with H&N MM were treated in 2005 with 57.6 Gy (RBE)/16 fractions/4 weeks and produced 2‐y OS, LC, and PFS rates of 50%, 100%, and 25%, respectively. Of these patients, 75% developed DM.^14^ Some patients with MM were treated using CIRT and chemotherapy. Three studies administered chemotherapy before, during, or after CIRT, and Demizu et al^16^ reported that 2‐y OS, LC, and PFS rates of 59%, 62%, and 41%, respectively. Recurrence was observed in 10.3% of the participants, and 40% of them developed DM. These outcomes were similar to those observed by Shirai et al,^18^ who found that concurrent administration of CIRT and DAV (dacarbazine; nimustine; vincristine) chemotherapy produced better PFS than CIRT alone; however, no significant difference between LC and OS rates was observed. In Italy, Vitolo et al^17^ reported eight cases suffering from inoperable malignant mucosal MMs in the upper aerodigestive tract, with macroscopic residuals/relapses following surgery or in patients that refused surgery; after CIRT, DM occurred in 25% of the patients. Mohr et al^19^ studied 18 cases of paranasal sinus MM, with 94% at stage IV, N (node) ≥1, and 17% with M (metastases). Of these patients, 89% received combined IMRT and CIRT, and the 3‐y OS and LC rates were 16.2% and 58.3%, respectively. Recurrence and metastasis occurred in 25% (four patients) and 44%, patients respectively.

**TABLE 3:** Main results of included studies

Studies	Tumor status	Treatment planning	Reported findings	Other therapies^c^	Local recurrence & DM
Zhang et al	Recurrences:100%	CTV: GTV + 0.5‐1.0 cm	1‐y OS/LC: 71.4% 3‐y OS: 71.4% 3‐y LC: 42.9%	None	Recurrence: 28.6%^a^ DM: 14.3%^a^
Mizoe et al^12^	No clear	CTV: GTV + enlarged lymph nodes	5‐y LC:100%	No clear	NR
Mizoe et al^13^	TxN0M0	CTV: GTV + enlarged lymph nodes	5‐y OS: 35% 5‐y LC: 75%	No clear	Recurrence: 2.4%^a^ DM: 41%^a^
Kagawa et al	T1: 12.5%; T2: 25% T3: 25%; T4: 37.5%	NR	2‐y OS:50% 2‐y LC: 100% 2‐y PFS: 25%	None	DM: 75%^a^
Demizu et al	T1: 35%; T2: 28% T3: 21%; T4: 17% Recurrences: 20% Initial treatment: 79%	CTV: GTV + 0.5 cm + adjacent cavity	2‐y OS: 62% 2‐y LC:59% 2‐y PFS: 41%	CT: 31% (DAV: before and/or after CIRT)	Recurrence: 10.3%^a^ DM: 40%^a^
Shirai et al	T3: 2.3%; T4: 97.7%	NR	2‐y OS/LC/PFS: 63%/88%/32%	Concurrent CT: 58% (DAV)	Recurrence: 9.3%^a^ DM: 53%^a^
Vitolo et al	Macroscopic residual following surgery: 37.5% postsurgical relapse with positive margins: 25%	NR	3‐month remission rate: 62.5%; 12‐month remission rate: 12.5%;	CT: 25% (before CIRT)	DM: 25%^a^
Toyama et al	T3: 93%; T4:7%	NR	5‐y OS: 80.4% 5‐y LC: 92.8%	None	Recurrence: 5.3%^a^ DM: 25.1%^a^
Mohr et al	T3: 6%; T4: 94% N ≥1:28%; M1: 17%; Recurrence: 50%	CTV1: macroscopic tumor and tumor bed; CTV2: CTV1 + spread and ipsilateral nodal	3‐y OS: 16.2% 3‐y LC: 58.3%	IMRT	Recurrence: 25%^a^ DM: 44%^a^
Barcellini et al	NR	Vaginal CTV1: inguinal lymph nodes + small pelvis (internal iliac, external iliac, obturator lymph nodes); CTV2: GTV + 5 mm	LC: median 10.23 m OS: median 11.41 m	None	Recurrence: 25%^a^ DM: 75%^a^
Murata et al	T1: 22%; T2: 56% T3: 22%; N ≥1:14% Recurrence: 32%; Initial treatment: 68%	CTV1: uterus, vagina and/or vulva, the pelvic lymph nodes and inguinal lymph nodes; PTV1:CTV1 + 5‐10 mm; CTV2:GTV + GTV node; PTV2:CTV2 + 5 mm;	5‐y OS 28% 5‐y LC 44%	None: 73% CT: 24%; Immunotherapy: 3%	DM: 45%^b^

Abbreviations: A, nimustine (ACNU); CT, chemotherapy; D, dacarbazine (DTIC); IMRT, intensity‐modulated radiotherapy; M, metastases; N, node; No clear, Data not available; T, tumor; V, vincristine (VCR).

^a^ Crude rates.

^b^ Cumulative rates.

^c^ Other therapies refer to treatments accompanied by CIRT, excluding the initial treatment regimen of recurrence patients.

In addition to the reports mentioned above, two retrospective studies reported diverse outcomes of CIRT used for treating gynecological MMs. The 5‐y OS and LC rates were 28% and 44%. DMs occurred in 45% and were more frequent in the younger age group (<71 years) than in older patients (52.9% vs 40.1%; *P* = .041).^22^ Barcellini et al^21^ reported short‐term therapeutic effects in four cases. Regardless of the small sample size, the prognosis of gynecological MMs appears to be worse than that of other MMs.

### 3.3 Toxicity

Acute and late cases of toxicity are shown in Table 4. Toxicity was low and acceptable in all studies. Out of six studies on CIRT used for treating H&N MMs, acute toxicity higher than G3 was not observed in four studies.^12^, ^14^, ^16^, ^17^ Shirai et al^18^ reported that acute reactions were not below G2, and they found no substantial difference in toxicity between CIRT with and without DAV therapy, apart from hematological adverse events. Only one study showed severe late toxicity with one patient showing G3 and one showing G4 reactions.^16^ In cases with paranasal sinus MM, G2 acute toxicity occurred in 17% of the patients and G3 in 28%. The severity of late toxicity was not above G2.^19^ None of the patients suffering from gynecological MM showed toxicity above G3.^21^, ^22^ In patients with choroidal MM, 31.6% developed neovascular glaucoma following CIRT.^15^

**TABLE 4:** Acute and late toxicities

Studies	Tumor site	Ages	Total dose (GyE)/fr	Toxicity
Zhang et al^a^	H&N: 28.6%; Limbs: 71.4%	No clear, but among 66 (33‐88)	61‐75/6‐11	Acute: ≤G3
Mizoe et al^12^, ^b^	Nasal and paranasal cavity/pharynx: 40% Middle ear: 20%	No clear, but among 60 (26‐77)	48.6‐70.2/16	Acute: ≤G3 Late: ≤G2
Mizoe et al^13^, ^b^	Nasal cavity: 54.1%; Paranasal sinus: 22.3% Oral cavity: 10.1%; Other: 13.1%	No clear, but among 56.5 (16‐80)	57.6 or 64/16 or 18	No clear
Kagawa et al^b^, ^c^	Nasal cavity: 62.5%; Ethmoid sinus: 25% Hard palate:12.5%	NR	57.6/16	Acute: G2: 62.5%; G3: 62.5% Late: G1: 62.5%;
Demizu et al^a^	Nasal cavity: 76%; Ethmoid sinus:10% Oral cavity:7%; Maxillary sinus:7%	72 (33‐89)	65 or 70.2/26	Acute: ≤G3 Late: G3: 3.4%; G4:3.4%
Shirai et al^a^	Nasal cavity: 81%	71 (32‐91)	57.6 or 64/16	Acute: ≥G2: 74% Late: ≥G2: 23%
Vitolo et al^a^	Nasal cavity: 50%; Oropharynx/oral cavity/nasopharynx/lacrimal duct: 12.5%	72 (48‐86)	68.8/16	Acute: G2: 87.5%; G3: 12.5% Late: ≤G1
Toyama et al	Choroid 100%	56(22‐83)	60‐85/16	Neovascular glaucoma: 31.6%
Mohr et al^a^	Paranasal sinus	68 (55‐80)	60‐74/16	Acute: G2:17%; G3: 28% Late: ≤G2
Barcellini 2019^a^	Vaginal:75%; Cervical:25%	60.5 (49‐72)	68.8/16 or 24/3	Acute: G1: 50%; G2:25%; G3: 25% Late: ≤G2
Murata 2019^a^, ^b^	Vaginal: 60% Vulval: 32% Cervical uterine: 8%	71 (51‐88)	57.6 or 64/16	Acute: G0: 75.6%; G1: 48.6%; G2: 37.8%; G3: 8.1% Late: G0: 75.6%; G1: 24.3%; G2: 10.8%

^a^ Toxicities were evaluated with the Common Terminology Criteria for Adverse Events version 3.0/4.0 (CTCAE v3.0/4.0).

^b^ Toxicities were scored according to Radiation Therapy Oncology Group/European Organization for Research and Treatment of Cancer (RTOG/EORTC).

^c^ Toxicities were scored according to NCI‐CTC 2.0.

## 4 DISCUSSION

We analyzed all available literature evidence of efficacy and safety of CIRT for treating MMs. The conclusions to be drawn from some of the publications may be limited due to certain caveats. Moreover, seven studies were retrospective. Application of CIRT for treating MMs is a currently developing research field, thus a systematic review of this small but heterogeneous body of evidence is needed and may be of use for further advance of application and knowledge in this field.

According to the RARECAREnet project (RARECAREnet, 2017), 1‐, 3‐, and 5‐y OS rates of MMs are 63%, 30%, and 20% in Europe.^23^ For definitive RT, the 5‐y OS rates ranged from 13% to 18%.^24^ A review also noted that no differences in 5‐y OS rates were observed between surgery plus radiation therapy and surgery only (25%‐46% vs 25%‐46.2%).^24^ Recently, definitive particle RT proved to be a promising treatment option, with 1‐/2‐year OS and LC rates of 91%/44% and 92%/71%, respectively, in patients treated with proton RT and 96%/62% and 95%/59%, respectively, in CIRT‐treated patients.^16^, ^24^ Proton RT or CIRT used for treating H&N MMs produced promising LC rates, whereas OS rates were unsatisfactory. This effect is most likely due to high rates of DM; however, the amount of available data are limited at present.

Compared with photons and protons, carbon‐ion beams directly cleave double‐stranded DNA at low oxygen concentrations and emit lower radiation doses to the surrounding healthy tissue, resulting in better therapeutic ratios. CIRT was first applied in MMs in 1994. Results showed 3‐y LC rates above 80%,^25^ even though 5‐y LC rates were 100% in locally advanced in cases with H&N mucosal MMs. Although conclusions may not be entirely robust due to the small sample sizes, high LC rates after CIRT treatments seem promising compared to other forms of H&N tumor treatments. In a clinical dose escalation trial, Mizoe et al observed 5‐y OS rates of 35% in 85 H&N mucosal MMs. Compared to CIRT, surgery‐only and postoperative photon RT produced similar 5‐y OS rates.^24^ However, considering that all studies included in this review reported low number of cases, it is difficult to conclude whether OS rates of these three treatment methods are truly similar. In general, tumors are typically localized at clinical presentation, and local infiltration and metastasis may occur at a later stage. Therefore, local control is the sine qua non for curative success. Results of the studies reviewed here suggest that CIRT can significantly increase LC rates, which is particularly important regarding small tumors. In 2017, Koto et al, retrospectively examined patients with H&N MM of N0‐1M0 status who had been CIRT‐treated at four Japanese institutions between 2003 and 2014.^26^ In total, 266 patients were enrolled in this multicenter study, and about half of the participants received chemotherapy. Two‐year LC and OS rates were 83.9% and 69.4%, respectively, and the 2‐y OS rate was significantly higher than those observed after other treatment methods (Table 5). In previous small‐sample studies, CIRT produced better LC rates but no distinctly improved survival benefit. In a large retrospective multicenter study, however, considerably improved OS rates were achieved, suggesting that failure to detect improved OS rates may be due to small sample sizes in other studies.

**TABLE 5:** Outcomes with various treatment methods for mucosal melanoma

Study	Year	No. of patients	Treatment	OS	LC
Plavc et al^67^	2016	48	Surgery + photon RT + systemic therapy	2‐y 43%	2‐y 52% (LRC)
Temam et al^53^	2005	39	Surgery + photon RT	2‐y 30%	2‐y 75%
Christopherson et al^59^	2015	5	Photon RT	5‐y 18%	—
Zenda e al^65^	2016	32	Proton RT	2‐y 55.9%	1‐y 75.8%
Nathan et al^68^	2019	63	Ipilimumab	18 m: 31.5%	—
Namikawa et al^69^	2018	12	Nivolumab + ipilimumab	2‐y 50%	—
Koto et al^26^	2017	266	CIRT	2‐y 69.4%	2‐y 83.9%

Abbreviation: LRC: local‐regional control.

Among the important clinicopathological factors of MMs, five independent predictors of mortality have been identified, and depth of primary tumors, termed T stage, is the most important factor. Metastasis is an important predictor of mortality.^27^ The majority of patients included in the studies of Kagawa et al, Shirai et al, and Mohr et al had T4 tumors, and the latter included 28% patients with N ≥1 and 17% M1. No significantly improved OS rates between CIRT and conventional RT seemed reasonable. Although including mostly patients at advanced stages, 2‐y OS rate reached 63% in the study of Shirai et al. All participants were N0 M0, and 58% used concurrent chemotherapy, which may explain the high survival rates. Koto et al suggested that concurrent chemotherapy was a markedly independent factor for OS,^26^ similar to results of concurrent chemo‐RT in patients with inoperable locally advanced nonsmall cell lung cancer.^28^ A recent study by Takayasu et al may have included the same patients as Shirai et al, therefore the former publication was not included in this review.^29^ They suggested that efficacy of DAV therapy combined with CIRT increased survival rates,^29^ however, also opposite results were reported.^30^ The patients included in the Shirai et al study were as well included in that of Koto et al; thus, further studies with larger sample sizes are required to determine whether concurrent chemotherapy is a predictor of OS rates. Distant metastases have worse prognosis, with median survival of 6‐9 months in untreated patients.^31^ Several metastatic MMs were included in the study of Mohr et al; therefore, they showed lower OS rates. Three reviewed studies reported PFS ranging from 25% to 41%, and CIRT concurrent with chemotherapy exhibited superior PFS compared to CIRT alone, which was also reported elsewhere.^28^, ^32^ In addition, T1‐2 tumors were significantly associated with better PFS. Recently, complementary activity of CTLA4 and PD‐1 checkpoint inhibitors in MM treatments has been demonstrated,^27^ and a combination of stereotactic body RT, anti‐CTLA‐4, and anti‐PD‐1 ligands promoted tumor responses in murine B‐16 models and patients.^33^ This suggests that CIRT and concurrent immunotherapy may help prolong survival in MMs.

Uveal MMs are characterized by high local control (90%), high metastasis rates (50%), and low survival. Nucleation, radiation with brachytherapy, and proton RT were the most common treatment methods.^34^ Tran et al reviewed clinical outcomes of choroidal MMs treated with proton beam therapy and 5‐y OS and LC rates were 85% and 91%, respectively.^35^ Verma et al also reviewed several results of proton RT for uveal MM treatments, and 5‐y OS and LC rates were 70%‐85% and above 90%, respectively,^36^ which were similar to those observed after iodine‐125 episcleral plaque RT (5‐y OS, LC, and DM of 84%, 91%, and 10%, respectively).^37^ Toyoma et al reported comparable OS and LC rates from CIRT for treating choroidal MMs at the NIRS.^15^ Notably, in their study, the incidence of neovascular glaucoma was 31.6%, and eye retention rates reached 91.8%.^38^ Accordingly, although differences between the two particle beams for treating superficial tumors are minimal, eye retention rates after CIRT were substantially higher than those after proton RT (74.3%). The cause of this was associated with reduced doses to the anterior segment and optic disc via 2‐port, which considerably contributed to decreasing incidences of neovascular glaucoma and increasing eye retention rates. In addition, small size of tumors, short tumor‐disc distance, and V_50_ of the iris‐ciliary body were also found to be associated with lower incidences of neovascular glaucoma.^15^ Taken together, carbon‐ion beams offer good dose consistency and biological effects, which may be of interest for patients with tumors close to critical structures of the eye. Numbers of patients and follow‐up periods in the reviewed studies may be insufficient for robust conclusions; however, CIRT appears to be a promising option for treating ocular MMs.

MMs are mostly of cutaneous origin; however, 18% of MMs occur in the female genital tract. The most common site of gynecological MMs is the vulva (70%), followed by the vagina and the lowest incidence in the cervix. In general, the prognosis of gynecological MM is poorer than that of non‐gynecologic MMs. Briefly, 5‐y OS were 18.8%, 11.1%, and 0% for stage I, stage II, and stages III‐IV, respectively.^39^, ^40^ Moreover, OS rates may vary between different MM sites. 5‐y OS rates were 37%‐50% in vulvar, 13%‐32% in vaginal, and 10% in cervical MM cases.^41^ Surgery, photon beam RT, chemotherapy, and PD‐1 checkpoint inhibitors were shown to not significantly improve OS rates^42^, ^43^; however, CIRT shows promising results for treating gynecological MMs. The NIRS was the first institution in the world to evaluate safety and efficacy of CIRT for treating gynecological MMs, and in 2009, the first publication on CIRT used for treating gynecological MMs was published. Six cases were treated using CIRT, and no severe toxicity or local recurrence was observed, but two patients developed DM. The study demonstrated that CIRT appears to be a safe and effective noninvasive choice for treating gynecological MMs.^44^ During the following 4 years, 17 patients were treated, and the outcomes of all 23 cases were evaluated in 2014. The 3‐y OS and LC rates and were 53.0% and 49.9%, respectively. However, large cervical MMs typically have substantially worse prognoses than those of vaginal and vulvar MMs. Although 61% patients developed recurrence, CIRT may still be a viable option as a conservative treatment.^45^ In 2019, Murata et al^22^ reported the long‐term outcomes of 37 cases treated at the NIRS. Five‐year OS, LC, and DM rates were 28%, 44%, and 57%, which were comparable to those observed in surgically treated patients.^46^ With the advance of CIRT, however, tumor disappearance was observed in 30 patients (81%). In addition, it is worth mentioning that the subjects included in this study were elderly patients with inoperable tumors and of a median age of 71 years. This suggests that gynecological MM patients treated using CIRT were in worse physical condition. In addition, 86.5% of the included patients had vaginal tumors, and 8% had cervical tumors. Of these patients, 78% had T2 or T3 and 14% showed lymph node metastases. By contrast, in a different study with similar survival rates, gynecological MM patients treated with surgery were younger than CIRT‐treated patients, with a median age of 56‐60 years. The vast majority of patients suffered from stage I vaginal tumors.^46^, ^47^ A case series report on CIRT for treating gynecological MMs in Italy also showed similar results. Prescription doses and fractionation schemes of one cervical MM and three vaginal MM cases were comparable to those observed at the NIRS. LC was 10.23 and 12.6 months in vaginal MM, and 7.3 months in cervical MM patients. Median OS was 11.41 months. Patients with lymph node metastases were also at a higher risk of relapse and death after 5 years.^21^, ^48^ Based on these results, CIRT also appeared to be applied safely and effectively in Italy. Patients in different geographical regions may produce different patterns of gene mutations and protein expression.^49^ Therefore, patients of diverse ethnicity and geographical distribution are needed for further research. Studies on CIRT and concomitant treatments with immune‐checkpoint inhibitors or targeted agents are also important.

During the early 21st century, CIRT treatments for various malignancies have been initiated in China, and patients with MM achieved promising survival rates.^20^ Only the study was available on patients with cutaneous MM on extremities, which is consistent with the epidemiology of MMs in China.^50^ All patients included in this study showed recurrent conditions, and the 3‐y OS rate was 71.4%. From this, it appears that patients with cutaneous MMs had better prognoses than those with mucosal MMs. A publication on epidemiology of genitourinary MMs suggested that cutaneous genitourinary MMs (penile or scrotal) were associated with better outcomes than mucosal genitourinary MMs (urothelial, vaginal, or vulvar).^51^ This hypothesis provided the basis for the abovementioned fact. Carvajal et al^52^ found that nodal metastases were less frequent in cutaneous MMs than in mucosal MMs, and patients with nodal diseases faced worse prognoses. Indeed, the lowest metastasis rate in all reviewed studies was 14.3%. Therefore, low metastasis rates are also one of the factors predicting high OS. Furthermore, cutaneous MMs develop at a younger age than mucosal MMs. Patients in good general condition are able to tolerate higher radiation doses, which may also indicate better prognoses. Briefly, CIRT was more effective for treating cutaneous MMs than for treating mucosal MMs. It should be noted, however, that there are large variations in prognoses due to differences between mucosal and cutaneous MMs regarding anatomical site, epidemiology, clinicopathologic characteristics, and genetics, even within the same ethnicity. Thus, further data are needed to draw robust conclusions.

It has been reported in several studies that 3.2%‐29% and 3%‐14% of patients with H&N MMs developed acute and late skin or mucosal toxicity of G3 or higher after photon, fast neutron, or proton RT, respectively. Especially, tumors close to critical anatomic structures show higher incidence of toxicity.^53^, ^54^, ^55^, ^56^ According to the reviewed studies on CIRT for H&N MM treatments, results indicated satisfactory dose localization characteristics of carbon‐ion beams. The maximum tolerance dose of skin to carbon ions was 70.2 Gy (RBE)/18 fractions/6 weeks and 64.0 Gy (RBE)/16 fractions/4 weeks. In all reviewed studies, G3 acute toxicity reached 28% in cases with paranasal sinus MM, which was similar to toxicities of photon/proton RT mentioned above. One possible explanation is that (clinical target volume) CTV2 included CTV1 (macroscopic tumor and tumor bed) plus typical pathways of spreading and ipsilateral nodal levels (II and III) as the median extended target volume in these case series was almost 337 mL, which was larger than volumes observed in other studies.^13^, ^16^, ^57^ It is generally believed that chemotherapy concurrent with RT increases RT toxicity.^58^ In the studies of Shirai et al and Vitolo et al, chemotherapy was used concurrent with RT in 58% and 25% of the patients, respectively, however, no significant differences in toxicity were observed between CIRT with and without DAV therapy, aside from hematologic adverse events. Therefore, chemotherapy concurrent with CIRT did not cause stronger toxicity. The grade and incidence of acute mucosal and skin adverse reactions after CIRT showed no significant differences from those observed after photon RT. Regarding late toxic effects, all reviewed studies reported only mild reactions: no case of high‐grade late toxicity was observed, aside from one patient who showed periodontal disease (G3) and one patient who developed nasal bleeding (G4); in contrast, Krengli et al and Christopherson et al reported fatal late complications in 6 out of 53 patients (11.3%) and in 3 out of 21 patients (14.3%) after surgery combined with conventional RT, respectively.^55^, ^59^ In all reviewed studies, although CIRT was associated with low rates of severe complications, acute toxicity remained high, which included predominantly mucositis and skin lesions. G1/2 acute and late toxicity were observed in 17%‐87.5% and in 10.8%‐62.5%, respectively, of all CIRT‐treated MMs, which were higher than those in patients treated with other conventional forms of RT.^55^, ^60^ Therefore, the mechanisms underlying CIRT causing toxicity in normal tissues close to tumors should be further investigated, and interventions should be applied to avoid unnecessary discomfort.

Tumor recurrence and metastasis are closely associated with the biological characteristics of tumor cells. Although surgery, RT, chemotherapy, immunotherapy, and targeted biological therapies are frequently used to treat MMs, they do not produce particularly strong effects with respect to controlling recurrence and metastasis. Local recurrence and DM rates were 29%‐60.7% and 29%‐76%, respectively, in patients treated with surgery only or with surgery plus postoperative RT^55^, ^59^, ^61^, ^62^ and 15%‐20% and 35%‐54.5%, respectively, after proton RT treatment.^16^, ^57^ Overall, CIRT presented comparatively lower recurrence (2.4%‐28.6%) but higher DM rates (14.3%‐75%). Regarding cutaneous MMs, CIRT is commonly used for treating recurrence patients, which may partly explain high recurrence rates (28.6%). However, only several studies included recurrence patients, and recurrence rate reporting was biased, which affects the robustness of our conclusions. Although carbon‐ion beams produce high linear energy transfer and RBE, which leads to DNA double‐strand breaks and prevents cell repair, DM rates in CIRT‐treated MM patients were comparably high. MMs are highly invasive tumors, however, although MMs were locally controlled, DMs occurred more frequently and earlier in patients with locally advanced tumors. It is worth noting that the majority of CIRT‐treated patients suffered from advanced MM. These facts may explain generally high metastases and poor OS rates. As is known, most MM patients carry one or more genetical mutations. The most prevalent mutations are in the genes *BRAF* (40%‐70%) and *NRAS* (15%‐30%); *C‐KIT* mutations showed the lowest prevalence (5%‐10%).^63^ Such mutations typically lead to aberrant activation of the RAS/RAF/MEK/ERK signaling cascades and of the phosphoinositol‐3‐kinase/AKT pathway, promoting tissue invasion and metastasis of MMs.^64^ Using targeted agents is therefore an important therapeutic strategy. Immunotherapy and kinase inhibitors are the backbone of modern systemic therapy for MMs, followed by chemotherapy. Of the patients in each of four studies, 25% and 50% received concurrent or adjuvant chemotherapy; however, this did not reduce local recurrence and DM rates, as was suggested by other researchers.^57^, ^65^ A study on gynecological MMs indicated that high DM rates may be due to high PD‐1 and/or PD‐L1 expression in patients.^66^ Therefore, concurrent or adjuvant use of anti‐PD‐L1/PD‐1 drugs or kinase inhibitors with CIRT should be examined in future clinical trials.

Our review had several limitations. Firstly, no relevant randomized clinical trial data are available due to present scarcity of carbon‐ion RT equipment and low incidence of MMs, while surgery remains the mainstay of treatment options. All studies reviewed here were clinical series reports with small sample sizes. Secondly, there is a bias in patient characteristics (such as TNM stage, tumor site, and application of other forms of therapy). In addition, OS, LC, toxicity, recurrence, and DM data were not consistently available because of incomplete follow‐up reporting. Furthermore, most patients included in the reviewed studies suffered from unresectable or relapsed tumors, whereas randomized controlled trials of other treatment modalities for comparable cases are lacking. These limitations therefore may affect reliability of the drawn conclusions. However, the following observations seem highly likely: (a) CIRT produces better LC with low recurrence and toxicity effects in MM treatments, compared to surgery, conventional photon RT, or proton RT; (b) despite better LC, CIRT still shows unsatisfactory OS rates. However, we believe that this therapy produces relatively good results, compared with those of other treatment options. This hypothesis is supported by the fact that almost all CIRT‐treated patients included in the reviewed studies had suffered from inoperable or recurrent tumors, and CIRT substantially reduced local recurrence. (c) In terms of OS after CIRT treatments, cutaneous and choroidal MMs showed better results than mucosal MMs. Among mucosal MMs, OS was lowest in patients with gynecological MMs. (d) CIRT concurrent with chemotherapy exhibited superior PFS compared to CIRT alone. (e) Patients younger than 71 years showed a higher incidence of DM during CIRT treatments of gynecological MMs. (f) Toxicity apparently did not differ between CIRT with and without DAV therapy, aside from hematologic adverse events. (g) Compared with surgery and conventional photon RT, CIRT seems to be a more promising option for patients with inoperable and recurrent tumors and tumors adjacent to important organs.

## 5 CONCLUSIONS

CIRT is a safe, effective, and feasible treatment option for MMs, particularly for patients with tumors close to critical anatomical structures or with recurrent tumors. Future efforts to improve prognoses should focus on both metastasis and survival. More effective systemic therapy options to be combined with CIRT are necessary.

## CONFLICT OF INTEREST

The authors have no conflict of interest.

## AUTHORS' CONTRIBUTIONS

Chengcheng Li finished the manuscript; Xiaohu Wang and Qiuning Zhang contributed to choose research directions. Zheng Li and Kehu Yang assessed the level of evidence; Ruifeng Liu and Hongtao Luo searched electronic database; Yichao Geng, Lina Wang, Shuangwu Feng, Xueshan Zhao and Zhen Yang contributed to screened literatures. Qiang Li revised the final manuscript.

## DECLARATION

The data used to support the findings of this study are available from the corresponding author upon request. No ethic approval was required as all data originated from previously published studies.

## Data Availability

I confirm that my article contains a Data Availability Statement even if no data is available (list of sample statements) unless my article type does not require one. I confirm that I have included a citation for available data in my references section, unless my article type is exempt.

## References

[1] Yanagi T , Mizoe J‐E , Hasegawa A , et al. Mucosal malignant melanoma of the head and neck treated by carbon ion radiotherapy. Int J Radiat Oncol Biol Phys. 2009;74(1):15‐20. 19046826 10.1016/j.ijrobp.2008.07.056

[2] Johansson M , Brodersen J , Gøtzsche PC , et al. Screening for reducing morbidity and mortality in malignant melanoma. Cochrane Database Syst Rev. 2019;6:CD012352. 31157404 10.1002/14651858.CD012352.pub2 PMC6545529

[3] Dummer R , Hauschild A , Lindenblatt N , et al. Cutaneous melanoma: ESMO Clinical Practice Guidelines for diagnosis, treatment and follow‐up. Ann Oncol. 2015;26(Suppl 5):v126‐v132. 26314774 10.1093/annonc/mdv297

[4] Espenel S , Vallard A , Rancoule C , et al. Melanoma: last call for radiotherapy. Crit Rev Oncol Hematol. 2017;110:13‐19. 28109401 10.1016/j.critrevonc.2016.12.003

[5] Ciren B , Wang X , Long Z . The evaluation of immunotherapy and chemotherapy treatment on melanoma: a network meta‐analysis. Oncotarget. 2016;7(49):81493‐81511. 27845904 10.18632/oncotarget.13277 PMC5348408

[6] Eggermont AM , Chiarion‐Sileni V , Grob JJ , et al. Adjuvant ipilimumab versus placebo after complete resection of high‐risk stage III melanoma (EORTC 18071): a randomised, double‐blind, phase 3 trial. Lancet Oncol. 2015;16(5):522‐530. 25840693 10.1016/S1470-2045(15)70122-1

[7] Petrella T , Verma S , Spithoff K , et al. Adjuvant interferon therapy for patients at high risk for recurrent melanoma: an updated systematic review and practice guideline. Clin Oncol (R Coll Radiol). 2012;24(6):413‐423. 22245520 10.1016/j.clon.2011.12.002

[8] Garbe C , Peris K , Hauschild A , et al. Diagnosis and treatment of melanoma. European consensus‐based interdisciplinary guideline‐update 2016. Eur J Cancer. 2016;63:201‐217. 27367293 10.1016/j.ejca.2016.05.005

[9] Kamada T , Tsujii H , Blakely EA , et al. Carbon ion radiotherapy in Japan: an assessment of 20 years of clinical experience. Lancet Oncol. 2015;16(2):e93‐e100. 25638685 10.1016/S1470-2045(14)70412-7

[10] Mizoe J , Hasegawa A , Takagi R , et al. Carbon ion radiotherapy for skull base chordoma. Skull Base. 2009;19(3):219‐224. 19881902 10.1055/s-0028-1114295 PMC2702197

[11] Moga C , Guo B , Schopflocher D , et al. Development of a Quality Appraisal Tool for Case Series Studies Using a Modified Delphi Technique. 2012 https://www.researchgate.net/publication/281411226_Development_of_a_Quality_Appraisal_Tool_for_Case_Series_Studies_Using_a_Modified_Delphi_Technique

[12] Mizoe J‐E , Tsujii H , Kamada T , et al. Dose escalation study of carbon ion radiotherapy for locally advanced head‐and‐neck cancer. Int J Radiat Oncol Biol Phys. 2004;60(2):358‐364. 15380567 10.1016/j.ijrobp.2004.02.067

[13] Mizoe J‐E , Hasegawa A , Jingu K , et al. Results of carbon ion radiotherapy for head and neck cancer. Radiother Oncol. 2012;103(1):32‐37. 22321201 10.1016/j.radonc.2011.12.013

[14] Kagawa K , Mayahara H , Oda Y , et al. Carbon ion radiotherapy for mucosal malignant melanoma of the head and neck. Int J Radiat Oncol Biol Phys. 2005;63(2):S356‐S356. 10.1016/j.ijrobp.2008.07.056 19046826

[15] Toyama S , Tsuji H , Mizoguchi N , et al. Long‐term results of carbon ion radiation therapy for locally advanced or unfavorably located choroidal melanoma: usefulness of CT‐based 2‐port orthogonal therapy for reducing the incidence of neovascular glaucoma. Int J Radiat Oncol Biol Phys. 2013;86(2):270‐276. 23414768 10.1016/j.ijrobp.2012.12.022

[16] Demizu Y , Fujii O , Terashima K , et al. Particle therapy for mucosal melanoma of the head and neck. A single‐institution retrospective comparison of proton and carbon ion therapy. Strahlenther Onkol. 2014;190(2):186‐191. 24362502 10.1007/s00066-013-0489-9

[17] Vitolo V , Fossati P , Bonora M , et al. Malignant mucosal melanoma in the upper aerodigestive tract treated with carbon ion RT at CNAO: preliminary results. Radiother Oncol. 2015;115:S728.

[18] Shirai K , Saitoh JI , Musha A , et al. Hypofractionated carbon‐ion radiation therapy for mucosal malignant melanoma in head and neck. Int J Radiat Oncol Biol Phys. 2017;99(2):E372. 10.1016/j.ijrobp.2017.04.032 28871995

[19] Mohr A , Chaudhri N , Hassel JC , et al. Raster‐scanned intensity‐controlled carbon ion therapy for mucosal melanoma of the paranasal sinus. Head Neck. 2016;38:E1445‐E1451. 26560744 10.1002/hed.24256

[20] Zhang H , Li S , Wang XH , et al. Results of carbon ion radiotherapy for skin carcinomas in 45 patients. Br J Dermatol. 2012;166(5):1100‐1106. 22136631 10.1111/j.1365-2133.2011.10764.x

[21] Barcellini A , Vitolo V , Facoetti A , et al. Feasibility of carbon ion radiotherapy in the treatment of gynecological melanoma. In Vivo. 2019;33(2):473‐476. 30804128 10.21873/invivo.11497 PMC6506293

[22] Murata H , Okonogi N , Wakatsuki M , et al. Long‐term outcomes of carbon‐ion radiotherapy for malignant gynecological melanoma. Cancers (Basel). 2019;11(4):482. 10.3390/cancers11040482 PMC6520847 30987391

[23] Ascierto PA , Accorona R , Botti G , et al. Mucosal melanoma of the head and neck. Crit Rev Oncol Hematol. 2017;112:136‐152. 28325255 10.1016/j.critrevonc.2017.01.019

[24] Lazarev S , Gupta V , Hu K , et al. Mucosal melanoma of the head and neck: a systematic review of the literature. Int J Radiat Oncol Biol Phys. 2014;90(5):1108‐1118. 25539369 10.1016/j.ijrobp.2014.03.042

[25] Okada T , Kamada T , Tsuji H , et al. Carbon ion radiotherapy: clinical experiences at national institute of radiological science (NIRS). J Radiat Res. 2010;51(4):355‐364. 20508375 10.1269/jrr.10016

[26] Koto M , Demizu Y , Saitoh JI , et al. Multicenter study of carbon‐ion radiation therapy for mucosal melanoma of the head and neck: subanalysis of the Japan Carbon‐Ion Radiation Oncology Study Group (J‐CROS) study (1402 HN). Int J Radiat Oncol Biol Phys. 2017;97(5):1054‐1060. 28332989 10.1016/j.ijrobp.2016.12.028

[27] Davar D , Kirkwood JM . Adjuvant therapy of melanoma. Cancer Treat Res. 2016;167:181‐208. 26601863 10.1007/978-3-319-22539-5_7 PMC8603200

[28] Hung MS , Wu YF , Chen YC . Efficacy of chemoradiotherapy versus radiation alone in patients with inoperable locally advanced non‐small‐cell lung cancer: a meta‐analysis and systematic review. Medicine (Baltimore). 2019;98(27):e16167. 31277121 10.1097/MD.0000000000016167 PMC6635168

[29] Takayasu Y , Kubo N , Shino M , et al. Carbon‐ion radiotherapy combined with chemotherapy for head and neck mucosal melanoma: prospective observational study. Cancer Med. 2019;8(17):7227‐7235. 31621203 10.1002/cam4.2614 PMC6885871

[30] Casalta‐Lopes J , Nobre‐Góis I , Teixeira T , et al. Neoadjuvant treatment in rectal cancer: long‐vs short‐course radiotherapy. Ann Oncol. 2012;23:ix208.

[31] Yu C , Liu X , Yang J , et al. Combination of immunotherapy with targeted therapy: theory and practice in metastatic melanoma. Front Immunol. 2019;10:990. 31134073 10.3389/fimmu.2019.00990 PMC6513976

[32] Chen W , Li T , Wang J , et al. Clinical study of nimotuzumab combined with concurrent radiochemotherapy for treatment of locally advanced cervical cancer. Cancer Manag Res. 2019;11:8157‐8165. 31564975 10.2147/CMAR.S191134 PMC6731987

[33] Twyman‐Saint Victor C , Rech AJ , Maity A , et al. Radiation and dual checkpoint blockade activate non‐redundant immune mechanisms in cancer. Nature. 2015;520(7547):373‐377. 25754329 10.1038/nature14292 PMC4401634

[34] Violanti SS , Bononi I , Gallenga CE , et al. New insights into molecular oncogenesis and therapy of uveal melanoma. Cancers (Basel). 2019;11(5):694. 10.3390/cancers11050694 PMC6562554 31109147

[35] Tran E , Ma R , Paton K , et al. Outcomes of proton radiation therapy for peripapillary choroidal melanoma at the BC Cancer Agency. Int J Radiat Oncol Biol Phys. 2012;83(5):1425‐1431. 22209152 10.1016/j.ijrobp.2011.10.017

[36] Verma V , Mehta MP . Clinical outcomes of proton radiotherapy for uveal melanoma. Clin Oncol (R Coll Radiol). 2016;28(8):e17‐e27. 26915706 10.1016/j.clon.2016.01.034

[37] Perez BA , Mettu P , Vajzovic L , et al. Uveal melanoma treated with iodine‐125 episcleral plaque: an analysis of dose on disease control and visual outcomes. Int J Radiat Oncol Biol Phys. 2014;89(1):127‐136. 24613808 10.1016/j.ijrobp.2014.01.026 PMC3988201

[38] Tsuji H , Ishikawa H , Yanagi T , et al. Carbon‐ion radiotherapy for locally advanced or unfavorably located choroidal melanoma: a Phase I/II dose‐escalation study. Int J Radiat Oncol Biol Phys. 2007;67(3):857‐862. 17161555 10.1016/j.ijrobp.2006.09.022

[39] Irvin WP , Legallo RL , Stoler MH , et al. Vulvar melanoma: a retrospective analysis and literature review. Gynecol Oncol. 2001;83(3):457‐465. 11733955 10.1006/gyno.2001.6337

[40] Pusceddu S , Bajetta E , Carcangiu ML , et al. A literature overview of primary cervical malignant melanoma: an exceedingly rare cancer. Crit Rev Oncol Hematol. 2012;81(2):185‐195. 21515070 10.1016/j.critrevonc.2011.03.008

[41] Wang L , Wang X , Zhang Q , et al. Is there a role for carbon therapy in the treatment of gynecological carcinomas? A systematic review. Future Oncol. 2019;15(26):3081‐3095. 31426679 10.2217/fon-2019-0187

[42] Garbe C , Eigentler TK , Keilholz U , et al. Systematic review of medical treatment in melanoma: current status and future prospects. Oncologist. 2011;16(1):5‐24. 21212434 10.1634/theoncologist.2010-0190 PMC3228046

[43] Kim MS , Choi C‐H , Kim T‐J , et al. Primary malignant melanoma of the uterine cervix treated with pembrolizumab after radical surgery: a case report and literature review. Obstet Gynecol Sci. 2018;61(4):524‐528. 30018908 10.5468/ogs.2018.61.4.524 PMC6046352

[44] Kiyohara H , Kato S , Ohno T , et al. Carbon ion radiotherapy for malignant melanoma of female genital organs. Int J Radiat Oncol Biol Phys. 2009;75(3‐supp‐S):S369.

[45] Karasawa K , Wakatsuki M , Kato S , et al. Clinical trial of carbon ion radiotherapy for gynecological melanoma. J Radiat Res. 2014;55(2):343‐350. 24536019 10.1093/jrr/rrt120 PMC3951082

[46] Frumovitz M , Etchepareborda M , Sun CC , et al. Primary malignant melanoma of the vagina. Obstet Gynecol. 2010;116(6):1358‐1365. 21099603 10.1097/AOG.0b013e3181fb8045

[47] Xia L , Han D , Yang W , et al. Primary malignant melanoma of the vagina: a retrospective clinicopathologic study of 44 cases. Int J Gynecol Cancer. 2014;24(1):149‐155. 24362720 10.1097/IGC.0000000000000013

[48] Khunger A , Buchwald ZS , Lowe M , Khan MK , Delman KA , Tarhini AA . Neoadjuvant therapy of locally/regionally advanced melanoma. Ther Adv Med Oncol. 2019;11:1758835919866959. 31391869 10.1177/1758835919866959 PMC6669845

[49] Palmieri G , Colombino M , Casula M , et al. Epidemiological and genetic factors underlying melanoma development in Italy. Melanoma Manag. 2015;2(2):149‐163. 30190844 10.2217/mmt.15.12 PMC6094587

[50] Huang YS , Chen XX , Yang SX , et al. Preliminary exploration of the clinical features of Chinese patients with skin malignancies and premalignancies: a retrospective study of 1420 cases from Peking University First Hospital. J Eur Acad Dermatol Venereol. 2013;27(9):1114‐1119. 22882540 10.1111/j.1468-3083.2012.04673.x

[51] Sanchez A , Rodríguez D , Allard CB , et al. Primary genitourinary melanoma: epidemiology and disease‐specific survival in a large population‐based cohort. Urol Oncol. 2016;34(4):166.e7‐166.e14. 10.1016/j.urolonc.2015.11.009 26739672

[52] Carvajal RD , Spencer SA , Lydiatt W . Mucosal melanoma: a clinically and biologically unique disease entity. J Natl Compr Canc Netw. 2012;10(3):345‐356. 22393195 10.6004/jnccn.2012.0034

[53] Temam S , Mamelle G , Marandas P , et al. Postoperative radiotherapy for primary mucosal melanoma of the head and neck. Cancer. 2005;103(2):313‐319. 15578718 10.1002/cncr.20775

[54] Lansu J , Klop WM , Heemsbergen W , et al. Local control in sinonasal malignant melanoma: comparing conventional to hypofractionated radiotherapy. Head Neck. 2018;40(1):86‐93. 29044881 10.1002/hed.24979

[55] Krengli M , Masini L , Kaanders JHAM , et al. Radiotherapy in the treatment of mucosal melanoma of the upper aerodigestive tract: analysis of 74 cases. A Rare Cancer Network study. Int J Radiat Oncol Biol Phys. 2006;65(3):751‐759. 16647223 10.1016/j.ijrobp.2006.01.016

[56] Liao JJ , Parvathaneni U , Laramore GE , et al. Fast neutron radiotherapy for primary mucosal melanomas of the head and neck. Head Neck. 2014;36(8):1162‐1167. 23852725 10.1002/hed.23428

[57] Fuji H , Yoshikawa S , Kasami M , et al. High‐dose proton beam therapy for sinonasal mucosal malignant melanoma. Radiat Oncol. 2014;9:162. 25056641 10.1186/1748-717X-9-162 PMC4118609

[58] He Y , Guo T , Guan H , et al. Concurrent chemoradiotherapy versus radiotherapy alone for locoregionally advanced nasopharyngeal carcinoma in the era of intensity‐modulated radiotherapy: a meta‐analysis. Cancer Manag Res. 2018;10:1419‐1428. 29922086 10.2147/CMAR.S160469 PMC5995285

[59] Christopherson K , Malyapa RS , Werning JW , et al. Radiation therapy for mucosal melanoma of the head and neck. Am J Clin Oncol. 2015;38(1):87‐89. 23563215 10.1097/COC.0b013e31828d73bf

[60] Bibault J‐E , Dewas S , Mirabel X , et al. Adjuvant radiation therapy in metastatic lymph nodes from melanoma. Radiat Oncol. 2011;6:12. 21294913 10.1186/1748-717X-6-12 PMC3041681

[61] Meleti M , Leemans CR , de Bree R , et al. Head and neck mucosal melanoma: experience with 42 patients, with emphasis on the role of postoperative radiotherapy. Head Neck. 2008;30(12):1543‐1551. 18704960 10.1002/hed.20901

[62] Bachar G , Loh KS , O'sullivan B , et al. Mucosal melanomas of the head and neck: experience of the Princess Margaret Hospital. Head Neck. 2008;30(10):1325‐1331. 18704964 10.1002/hed.20878

[63] Moltara ME , Novakovic S , Boc M , et al. Prevalence of BRAF, NRAS and c‐KIT mutations in Slovenian patients with advanced melanoma. Radiol Oncol. 2018;52(3):289‐295. 30210039 10.2478/raon-2018-0017 PMC6137366

[64] Leonardi G , Falzone L , Salemi R , et al. Cutaneous melanoma: From pathogenesis to therapy (Review). Int J Oncol. 2018;52(4):1071‐1080. 29532857 10.3892/ijo.2018.4287 PMC5843392

[65] Zenda S , Akimoto T , Mizumoto M , et al. Phase II study of proton beam therapy as a nonsurgical approach for mucosal melanoma of the nasal cavity or para‐nasal sinuses. Radiother Oncol. 2016;118(2):267‐271. 26547102 10.1016/j.radonc.2015.10.025

[66] Hou JY , Baptiste C , Hombalegowda RB , et al. Vulvar and vaginal melanoma: a unique subclass of mucosal melanoma based on a comprehensive molecular analysis of 51 cases compared with 2253 cases of nongynecologic melanoma. Cancer. 2017;123(8):1333‐1344. 28026870 10.1002/cncr.30473

[67] Plavc G , But‐Hadžić J , Aničin A , et al. Mucosal melanoma of the head and neck: a population‐based study from Slovenia, 1985–2013. Radiat Oncol. 2016;11(1):137. 27737700 10.1186/s13014-016-0712-9 PMC5064955

[68] Nathan P , Ascierto PA , Haanen J , et al. Safety and efficacy of nivolumab in patients with rare melanoma subtypes who progressed on or after ipilimumab treatment: a single‐arm, open‐label, phase II study (CheckMate 172). Eur J Cancer. 2019;119:168‐178. 31445199 10.1016/j.ejca.2019.07.010

[69] Namikawa K , Kiyohara Y , Takenouchi T , et al. Efficacy and safety of nivolumab in combination with ipilimumab in Japanese patients with advanced melanoma: an open‐label, single‐arm, multicentre phase II study. Eur J Cancer. 2018;105:114‐126. 30447539 10.1016/j.ejca.2018.09.025

